# Serum levels of kisspeptin are elevated in critically ill patients

**DOI:** 10.1371/journal.pone.0206064

**Published:** 2018-10-17

**Authors:** Mark Luedde, Martina E. Spehlmann, Hans-Joerg Hippe, Sven H. Loosen, Sanchari Roy, David Vargas Cardenas, Mihael Vucur, Norbert Frey, Alexander Koch, Tom Luedde, Christian Trautwein, Frank Tacke, Christoph Roderburg

**Affiliations:** 1 Department of Internal Medicine III, University Clinic of Schleswig-Holstein, Kiel, Germany; 2 Department of Medicine III, University Hospital RWTH Aachen, Aachen, Germany; Medical University Graz, AUSTRIA

## Abstract

**Introduction:**

Members of the adipokine family such as resistin, adiponectin and omentin have recently been described as novel biomarkers with a diagnostic and prognostic role in the context of critically ill patients during intensive care unit (ICU) treatment. Kisspeptin represent another member of this family and has been shown to be closely correlated to different members of the adipokine family in manifold diseases. However, its role in critical illness and sepsis is currently unknown.

**Materials and methods:**

Kisspeptin serum concentrations were measured in 133 ICU patients admitted to the medical ICU. Results were compared with 36 healthy controls.

**Results:**

Kisspeptin serum levels were elevated in the serum of critically ill patients at admission to the ICU, when compared to healthy controls, and remained increased after 72 hours of ICU treatment. Notably, kisspeptin levels were independent of the presence of sepsis and etiology of critical illness. In line, serum concentrations of kisspeptin were not correlated to concentrations of inflammatory cytokines or established sepsis markers. Serum kisspeptin correlated inversely with the glomerular filtration rate. In contrast to the reported role of other members of the adipokine family, serum levels of kisspeptin were neither predictive for short term survival during ICU treatment nor for patients’ overall survival. Kisspeptin levels did not correlate with other adipokines measured in serum, including leptin, resistin, ghrelin, or adiponectin.

**Conclusions:**

Although circulating kisspeptin levels were strongly elevated in ICU-patients, elevated kisspeptin levels were not predictive for an impaired patients' survival.

## Introduction

Recent studies have demonstrated that the visceral adipose tissue not only holds crucial roles as an endocrine organ maintaining homeostasis [1, 2] but also plays a pivotal role in different pathological situations, such as cardiovascular diseases [3, 4], rheumatioid arthritis [5], and also metabolic conditions like obesity [6].

Among those mediators of the adipose tissue (so-called adipokines), kisspeptin has been described in various physiological and pathological settings. First discovered in 1996 in melanoma cells, Lee et al. were able to detect high levels of kisspeptin-mRNA mainly in the placenta [7]. Moreover, an important function of kisspeptin was described in the hypothalamus-pituitary-gonadal axis [8–10]. However, recent discoveries revealed that the kisspeptin receptor GPR45 is expressed in various other tissues, such as pancreas, liver and skeletal muscle [11], hinting towards a crucial role of kisspeptin as an adipokine involved in energy metabolism and homeostasis. However, not only physiological and sexual development are areas directly influenced by kisspeptin. As it is the case for adipokines in general, dysregulation of kisspeptin during different diseases seems to be relevant in the pathogenesis of various illnesses. As might be expected, metabolic disorders in general and obesity specifically show an impaired kisspeptin-signaling [12], but also inflammatory situations are associated with activated kisspeptin [13, 14].

Based on these considerations, we and others hypothesized that adipokines can serve as biomarkers for severe conditions, such as septic disease [15–19]. In this work, we addressed the potential clinical and prognostic relevance of kisspeptin in critical illness and/or sepsis. We evaluated circulating kisspeptin levels longitudinally in 133 medical ICU patients. Although our data showed a significant increase in circulating kisspeptin levels, there was limited value for assessing disease severity, organ failure or mortality in the patients admitted to the ICU.

## Materials and methods

### Study design and patient characteristics

133 patients that were consecutively admitted to our medical ICU were included into the study. The cohort consisted of 75 male and 58 female patients, respectively. Patient characteristics are shown in Table 1. The study protocol was approved by the local ethics committee (ethics committee of the University Hospital Aachen, RWTH-University, Aachen, Germany) and conducted in accordance with the ethical standards laid down in the Declaration of Helsinki. Written informed consent was obtained from the patient, his or her spouse, or the appointed legal guardian. Patient information and samples were acquired prospectively, and follow-up was performed as recently described [13]. Blood from healthy controls was obtained from the local blood bank after written informed consent. Presence of septic disease was defined according to the criteria defined in the third consensus definition of sepsis [14]. All other patients were categorized as non-septic patients [15, 16]. Kisspeptin serum levels in critically ill patients were compared with 36 healthy blood donors.

**Table 1:** Study population.

Parameter	all patients
Number	133
Sex (male/female)	75 / 58
Age median (range) [years]	66 (18–90)
APACHE-II score median (range)	19 (3–40)
SAPS2 score median (range)	44 (9–68)
ICU days median (range)	9 (1–137)
Death during ICU (%)	26.3%
Ventilation time median (range) [h]	121 (0.0–2966.0)
pre-existing diabetes n(%)	33.8%
suPAR ng/ml	9.8 (0.0–20.0)
BMI [kg/m^2^]	25.99 (15.9–59.5)
WBC median (range) [x10^3^/μl]	12.7 (0.1–208)
CRP median (range) [mg/dl]	126 (<5–230)
Procalcitonin median (range) [μg/l]	1.0 (0.0–125.2)
Interleukin-6 median (range) [pg/ml]	73 (0–6100)

APACHE, Acute Physiology and Chronic Health Evaluation; CRP, C-reactive protein; ICU, intensive care unit; SAPS, simplified acute physiology score; WBC, white blood cell count

### Measurements of kisspeptin serum levels by ELISA

Blood samples were collected upon admission to the ICU (prior to therapeutic interventions) as well as in the morning of Day 3 after admission. After immediate centrifugation at 2,000 g at 4°C for 10 minutes, serum and plasma aliquots of 1 mL were frozen immediately at -80°C in order to avoid repetitive freeze-thaw cycles as described previously [15, 17, 18]. We determined kisspeptin serum levels by using a commercially available enzyme-linked immunosorbent assay (Human Kisspeptin 1 (KISS1) ELISA Kit from Abbexa (cat. nr. abx152134)) according to manufacturers’ instructions.

### Statistical analysis

Statistics applied in this analysis have been described recently [15, 17, 18]. In summary, data are expressed as median and range. The Mann-Whitney- *U*-test and for multiple comparisons the Kruskal-Wallis-ANOVA were used. Box plot graphics display a statistical summary of the median, quartiles, ranges, and extreme values. Correlation analysis was performed by using the Spearman correlation test, and the prognostic value of the variables was tested by univariate and multivariate analysis in the Cox regression model. Kaplan-Meier curves were plotted to display the impact on the patients’ survival. Finally, ROC curves were generated by plotting sensitivity against 1-specificity. All statistical analyses were performed with SPSS (SPSS, Chicago, IL, USA) [19, 20].

## Results

### Kisspeptin serum concentrations in critically ill patients

**Fig 1:**
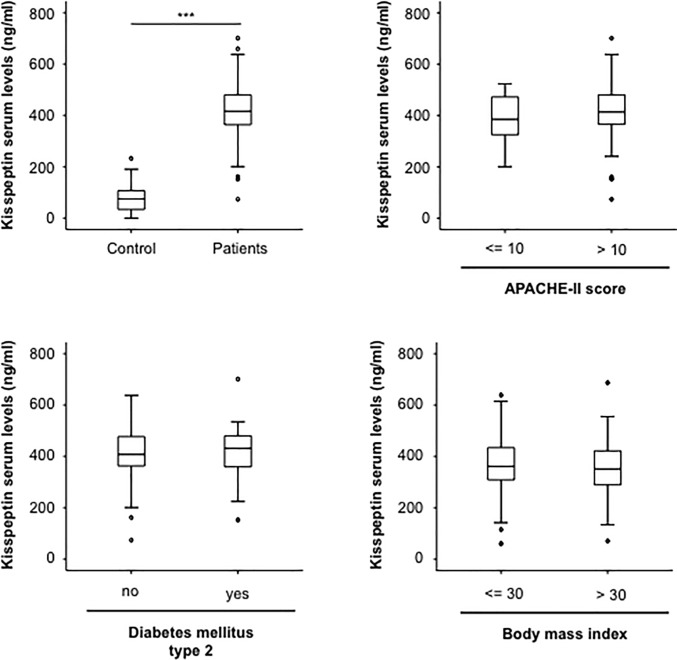
Kisspeptin serum concentrations in critically ill patients. (a) Serum levels of kisspeptin levels significantly elevated in critically ill patients at the time-point of admission to the ICU when compared to healthy controls. (b) Serum levels of kisspeptin were independent of the disease severity, as shown by similar kisspeptin concentrations in patients with high or low APACHE-II scores. (c) Kisspeptin levels were similar in patients with or without type 2 diabetes. (d) Kisspeptin serum levels were similar in patients with or without obesity (defined as body mass index [BMI] above 30 kg/m^2^). *** p < 0.001.

We have recently demonstrated that serum levels of different adipokines are elevated in critically ill patients treated on a medical ICU [15–19]. Based on these data we now aimed to analyze a potential role of kisspeptin, another member of the adipokine family, as a serum marker in critical illness and sepsis. We measured serum levels of kisspeptin in a large and well defined cohort of 133 ICU-patients and compared them to those measured in 36 healthy blood donors as a control (patient characteristics are given in Table 1). In this analysis, kisspeptin concentrations were significantly higher in the patients´ group compared to controls (Fig 1A). We next compared kisspeptin serum levels between patients with a more severe critical illness according to higher APACHE-II scores to patients with less severe disease state. Unexpectedly, no difference was found between these subgroups of patients (Fig 1B). Alterations in kisspeptin levels were demonstrated in different metabolic diseases [20, 21]. We therefore analyzed whether obesity and type 2 diabetes mellitus might have an influence on kisspeptin levels also in patients with critical illness. However, in our cohort of critically ill medical patients, kisspeptin serum concentrations were not affected by the presence of these metabolic comorbidities (Fig 1C and 1D). Moreover, by Spearman rank correlation analysis no correlation between kisspeptin and serum glucose levels or BMI was present (Table 2; Fig 1E and 1F). Finally, kisspeptin concentrations were independent on the patient´s age or sex (not shown).

**Table 2:** Correlations of Kisspeptin serum concentrations and other laboratory markers at day of admission to the ICU.

	ICU admission		ICU day 3	
Parameter	r	p	r	p
				
***Markers of liver function***				
Albumin	-.063	0.565	-0.106	0.390
INR	-0.045	0.610	0.078	0.466
AST	0.068	0.467	0.141	0.206
ALT	-0.039	0.655	-0.017	0.872
Bilirubin	-0.003	0.977	0.081	0.453
GLDH	0.070	0.464	-0.031	0.785
***Markers of inflammation***				
CRP	0.045	0.606	0.085	0.431
** Procalcitonin**	0.204	0.059	0.325	**0.005**
** IL-10**	**0.281**	**0.041**	0.129	0.361
IL-6	-0.071	0.477	0.054	0.668
TNF	0.102	0.522	0.071	0.657
** suPAR**	**0.235**	**0.008**	**0.214**	**0.043**
***Markers of renal function***				
Creatinine	-0.247	0.146	0.054	0.756
** Urea**	**0.157**	**0.041**	**0.259**	**0.014**
** GFR**	**-0.237**	**0.026**	-0.234	0.064
** Cystatin C**	**0.364**	**0.003**	**0.427**	**0.001**
** GFR with Cystatin C**	**-0.348**	**0.006**	**-0.335**	**0.009**
***Metabolic markers***				
Adiponectin	-0.010	0.938	0,096	0.475
Leptin	0.060	0.659	0.125	0.359
Leptin receptor	0,007	0.959	0.146	0.277
Ghrelin	-0.101	0.452	-0.088	0.517
BMI	-0.016	0.859	0.067	0.552
** HbA1c**	**0.287**	**0.021**	0.103	0.421
Insulin	-0.033	0.795	0.287	0.023

r, correlation coefficient; p, p-value; r and p-values by Spearman rank correlation

### Kisspeptin serum concentrations in patients with sepsis

**Fig 2:**
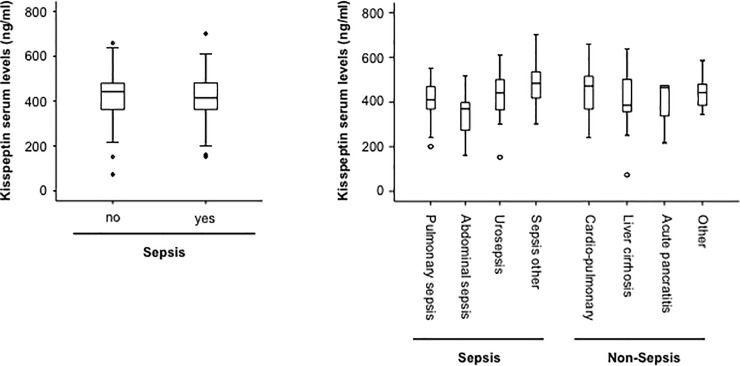
Kisspeptin serum concentrations in patients with sepsis. (a) ICU patients with sepsis (n = 94) displayed similar kisspeptin serum levels compared to patients that did not fulfill the sepsis-3 criteria (n = 39, U-test). (b) Levels of circulating kisspeptin were not altered in patients with different etiologies of critical illness.

Within our cohort of 133 critically ill patients, 94 fulfilled the sepsis-3 criteria, while 39 were admitted to the ICU due to other causes of critical illness (Table 3). No differences in kisspeptin concentrations between patients´ with or without sepsis were found (Fig 2A). In line, kisspeptin levels did not correlate with established sepsis-markers routinely assessed in critically ill patients such as C-reactive protein (CRP), procalcitonin (PCT) and interleukin-6 (IL-6) (Table 2). In contrast, kisspeptin levels correlated with suPAR serum levels, an experimental SIRS-marker [22] and serum levels of IL-10 as an anti-inflammatory cytokine. Interestingly, when extending this analysis on other adipokines, only Resistin demonstrated a similar correlation to IL-10 (S1 Table). Among the 94 patients fulfilling the sepsis-3 criteria, 54 suffered from pulmonary sepsis, 12 from abdominal sepsis, 6 from urogenital infections, and 22 from septic diseases with a different or unknown focus. By comparing concentrations of circulating kisspeptin between these different groups, we did not observe any substantial differences (Fig 2B). In line to these data, expression levels of kisspeptin were not altered in neutrophils extracted from patients with or without septic disease (*data from GSE6535 [23] and GSE5772 [24])*, highlighting that white blood cells do not represent and major source of serum-kisspeptin. Among the non-septic patients, 15 suffered from cardio-pulmonary diseases, 9 from decompensated liver cirrhosis and 11 had another etiology of critical illness. Again, by comparing concentrations of circulating kisspeptin between these different groups, we did not observe any substantial differences (Fig 2B). Since recently lowered kisspeptin levels were reported in patients with acute myocardial infarction, we compared kisspeptin serum concentrations of patients with known coronary artery disease (cad) with those of patients without cad. However also in this analysis no differences became apparent (S2 Fig).

**Table 3:** Disease etiology of the study population.

	sepsis	non-sepsis
	94	39
**Etiology of sepsis critical illness** Site of infection n (%)		
Pulmonary	54	
Abdominal	12	
Urogenital	6	
Other	22	
**Etiology of non-sepsis critical illness** n (%)		
cardiopulmonary disease		15
decompensated liver cirrhosis		9
acute pancreatitis		4
non-sepsis other		11

In summary, these data demonstrate that kisspeptin serum concentrations are not useful as a general marker to distinguish critically patients with sepsis from those with a non-septic disease etiology.

### Alterations in Kisspeptin serum levels during the early course of ICU treatment

**Fig 3:**
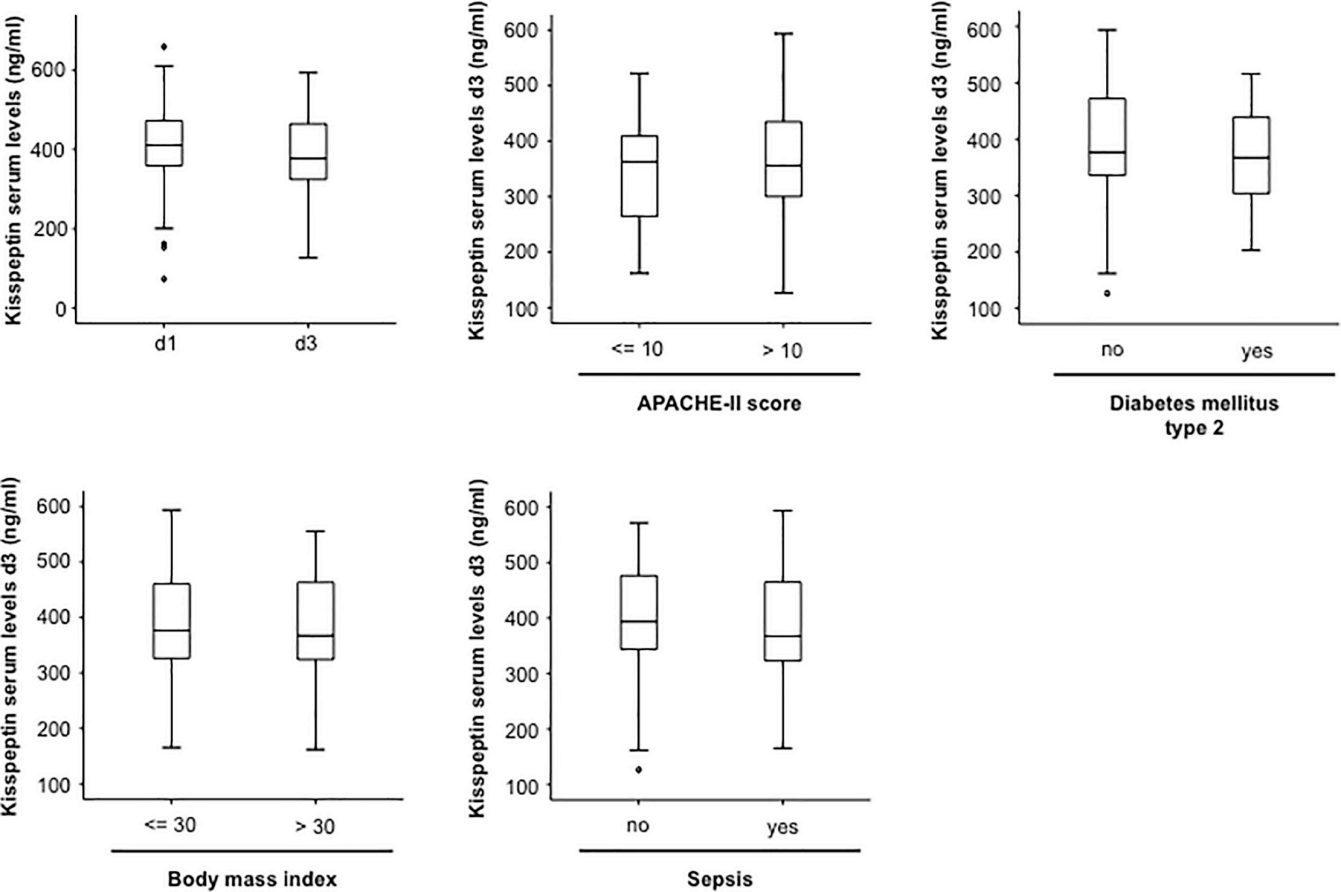
Alterations in Kisspeptin serum levels during the early course of ICU treatment. (a) Serum concentrations of kisspeptin measured at the time-point of admission to the ICU and three days of ICU-treatment. (b) Serum levels of kisspeptin after 3 days of ICU-treatment were independent of the disease severity according to similar kisspeptin concentrations in patients with high/ low APACHE-II scores. (c) Kisspeptin levels after 3 days of ICU-treatment were similar in patients with or without type 2 diabetes. (d) Kisspeptin serum levels after 3 days of ICU-treatment were similar in patients with or without obesity. (e) Kisspeptin serum levels after 3 days of ICU-treatment were similar in patients with or without sepsis.

Longitudinal measurements were available for 90 out of 133 patients. Of note, kisspeptin concentrations at day 3 of ICU treatment were significantly lower than that found at admission to the ICU, but still significantly higher compared to healthy controls (Fig 3A, not shown). In line to the results found when kisspeptin concentrations were measured at admission to the ICU, kisspeptin concentrations at day 3 were not related to disease severity (Fig 3B), the presence of metabolic diseases (Fig 3C and 3D) or the presence of septic diseases (Fig 3E), respectively.

### Elevated kisspeptin concentration do not reflect patients survival

**Fig 4:**
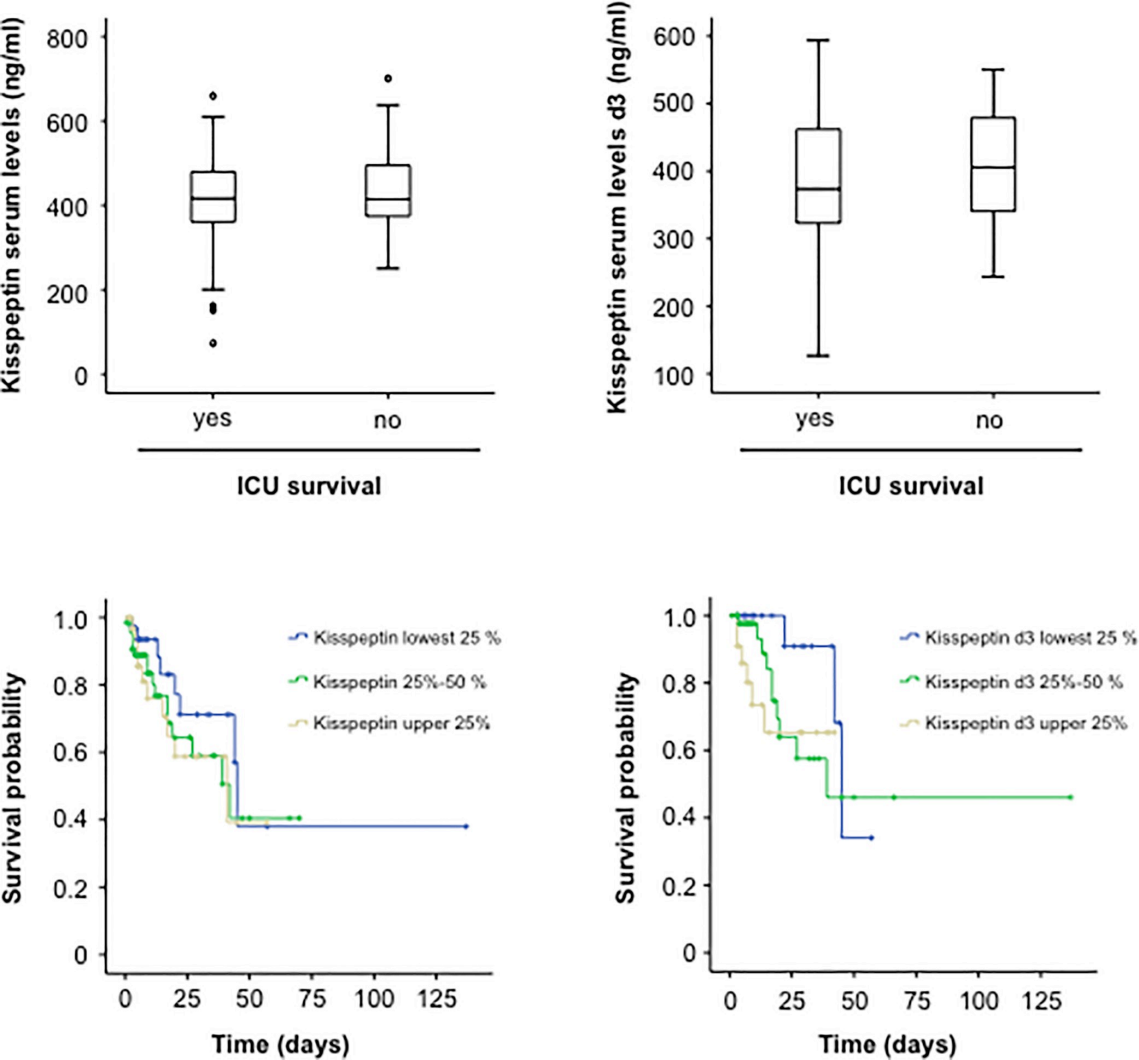
Elevated kisspeptin concentrations do not reflect ICU-survival. (a) Serum levels of kisspeptin at admission to the ICU were similar in patients that survived ICU treatment and patients that succumbed to death during ICU treatment. (b) Serum levels of kisspeptin after three days of ICU-treatment were similar in patients that survived ICU treatment and patients that succumbed to death during ICU treatment. (c) Kaplan-Meier curve analysis revealing that serum levels of kisspeptin at admission to the ICU did not reflect patients ICU survival. (d) Kaplan-Meier curve analysis revealing that serum levels of Kisspeptin after three days of ICU treatment did not reflect patients ICU survival.

Recent studies, including our own investigations [15–19], have demonstrated a strong association between elevated adipokine concentrations and the mortality risk in patients with systemic inflammatory diseases. We therefore we compared kisspeptin serum concentrations both at admission and after three days of ICU treatment between ICU survivors and patients that did not survive. In contrast to other adipokines, there was no difference between patients that survived and patients that died (Fig 4A and 4B). In line, Kaplan Meier curve analyses revealed no differences in patient survival between patients with higher or lower kisspeptin concentrations (Fig 4C and 4D).

**Fig 5:**
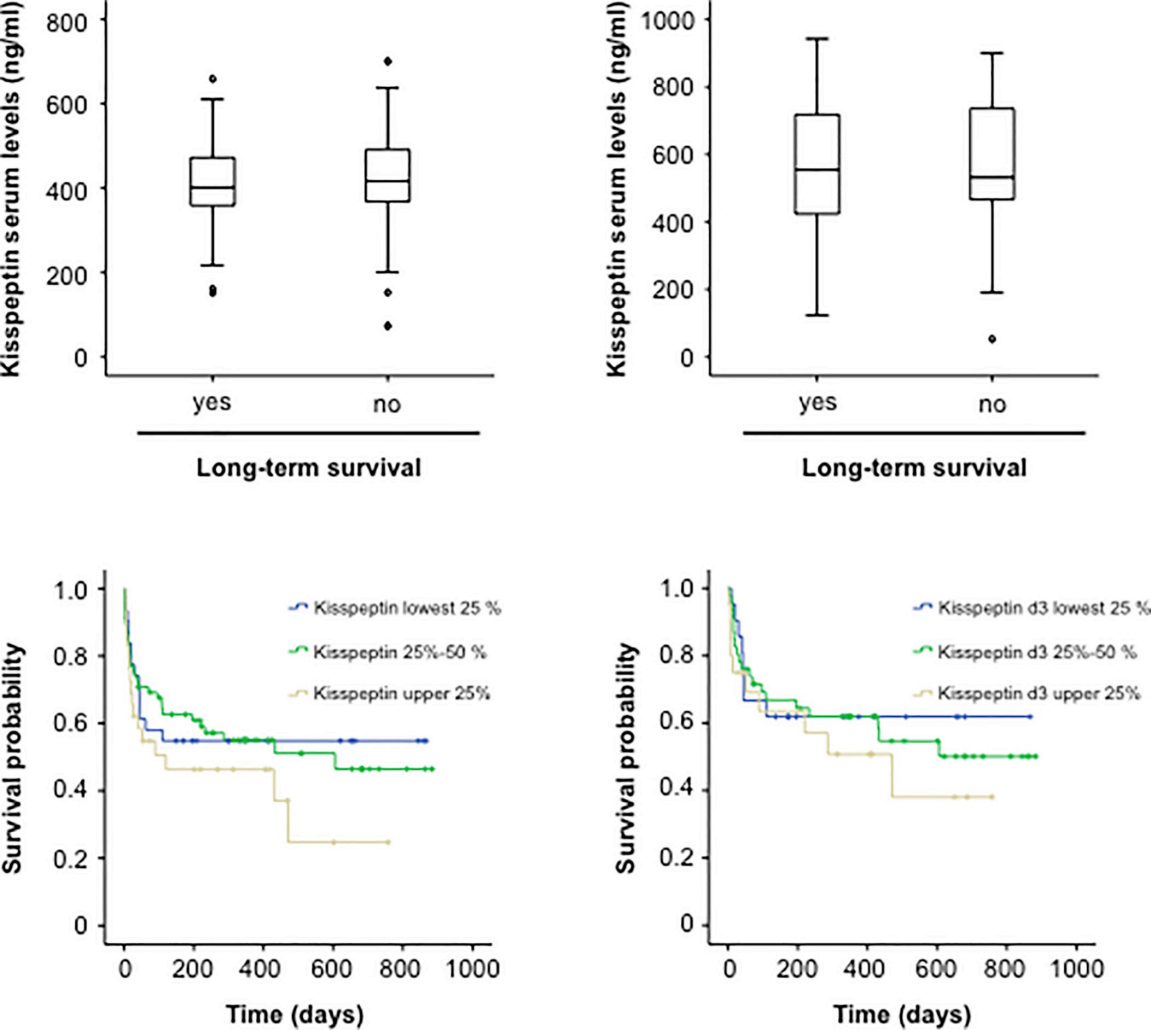
Elevated kisspeptin concentrations do not reflect long-term survival. (a) Serum levels of kisspeptin at admission to the ICU were similar in patients that survived in the long-term observation period and patients that succumbed to death. (b) Serum levels of kisspeptin after three days of ICU-treatment were similar in patients that survived in the long term observation period and patients that succumbed to death. (c) Kaplan-Meier curve analysis revealing that serum levels of kisspeptin at admission to the ICU did not reflect patients long-term survival. (d) Kaplan-Meier curve analysis revealing that serum levels of kisspeptin after three days of ICU treatment did not reflect patient long-term survival.

Within our cohort of critically ill patients, 26.3% died on the ICU and an additional 22.1% died during long-term follow up. We therefore tested whether kisspeptin levels at admission to ICU and after three days of ICU treatment were predictive for the patients’ long-term prognosis. However, also in this analysis we could not detect a difference in kisspeptin concentrations between patients that survived during the long-term follow up period and those patients that succumbed to death (Fig 5A and 5B). Finally, we again performed Kaplan-Meier curve analyses to determine the impact of elevated kisspeptin levels on the overall patients’ survival in our cohort of critically ill patients. Kisspeptin levels were not indicative for patients´ long term survival in this analysis (Fig 5C and 5D).

### Serum levels of kisspeptin correlate with markers of kidney injury

**Fig 6:**
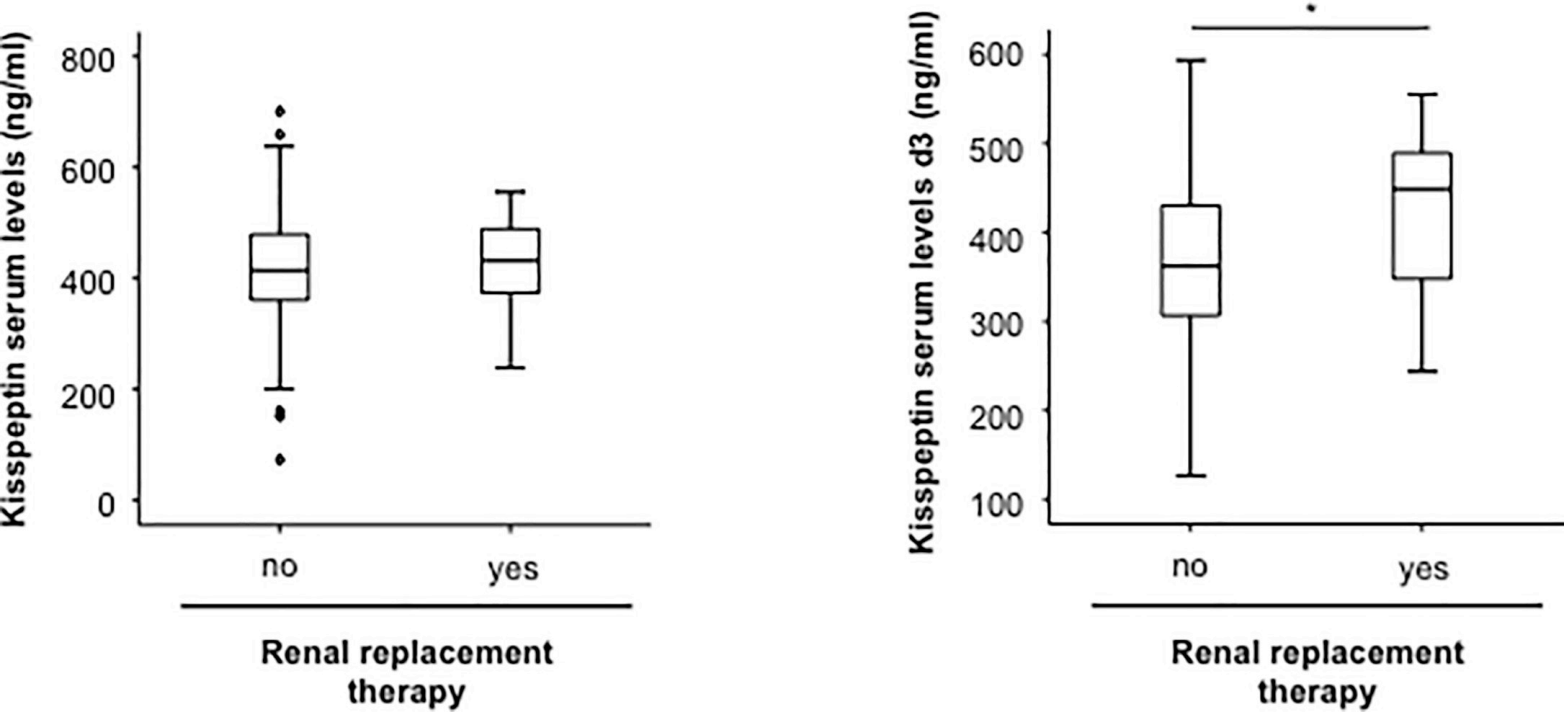
Alterations in kisspeptin serum levels in patients with renal replacement therapy. (a) Serum concentrations of kisspeptin measured at the time-point of admission to the ICU in patients with or without need for renal replacement therapy. (b) Serum concentrations of kisspeptin measured at d3 in patients with or without need for renal replacement therapy.

In order to identify factors determining kisspeptin serum levels in patients with critical illness, we next applied correlation analyses between kisspeptin serum concentrations and a broad set of clinically and experimentally measured laboratory parameters. While kisspeptin serum levels did not correlate with markers of organ failure or traditional prognostic ICU scores (Table 3), we found a strong correlation to parameters indicating a decreased renal function such as the glomerular filtration rate (GFR) and serum creatinine (Table 3), suggesting the renal elimination of kisspeptin. Based on these findings, we analyzed whether patients with a need for a renal replacement therapy (RRT) displayed different kisspeptin levels compared to patients with a preserved kidney function. Interestingly, RRT-patients displayed significantly elevated kisspeptin levels at d3, which is most likely due to a retention of kisspeptin specifically in patients with renal failure (Fig 6). Finally, we attempted to identify a correlation between kisspeptin levels and other adipokines. However, no such correlations could be established, highlighting that different adipokines might fulfill different functions or reflect different processes with a prognostic role in the context of critical illness (Table 3).

## Discussion

Biomarkers are a growing research interest in translational medicine. The use of biomarkers holds a vast potential for diagnostic and prognostic objectives in postoperative and critically ill patients, who display diverse degrees of inflammation, infection, and accompanying organ dysfunction or failure. Kisspeptin is a peptide expressed in humans mainly by neurons of the hypothalamus. Furthermore, expression in the testicles and uterus has been demonstrated [25, 26]. Kisspeptin is an essential part of the hypothalamic signaling pathway [27, 28]: it commences puberty in both non-primate and primate vertebrates by communication via the kisspeptin receptor [29, 30] and sustains the major function of the reproductive system in the adult [27, 31, 32]. Of note, kisspeptin is expressed by tumor cells and carries out pivotal tasks in tumor suppression [33]. To clarify the localization of kisspeptin production in sepsis, we analyzed two publicly available datasets of kisspeptin mRNA expression levels in neutrophils and found that kisspeptin mRNA expression did not differ between patients with and without septic disease. There are currently no reports on significant expression of the kisspeptin peptide in neutrophils. We therefore presume that the major cellular source of kisspeptin are not the white blood cells. Since neuropeptides are enzymatically inactivated [34] the half-life of the different kisspeptin isoforms is short (3.8 minutes for kisspeptin 10 [35] and 27.6 minutes for kisspeptin 54 [31]).

Our study describes for the first time that circulating kisspeptin distinguishes critically ill patients from healthy probands at the timepoint of admission to the ICU and after 72 hours of intensive care unit treatment. Our data rely on a large consecutively recruited cohort that comprises a broad spectrum of critically ill medical patients that were precisely characterized regarding clinical characteristics including severity of illness, as exemplarily defined by the APACHE II score. Although there are so far no known studies describing the association of kisspeptin serum levels and critical illness, a recent meta-analysis demonstrated that remarkable changes of circulating adipokines have been detected in critically ill patients and that some of these adipokine levels could predict patient outcomes [36]. Adipokines represent a substantial sum of hormones and cytokines that are secreted by the endocrine organ of the adipose tissue. In this study, elevated levels of circulating resistin and visfatin are linked to unfavorable outcomes of critically ill patients including more severe inflammation and higher risk of organ dysfunction and mortality [36]. However, in our study kisspeptin levels were neither predictive for short term survival during ICU treatment nor were they predictive for the overall patient survival.

Our study shows a significant increase in kisspeptin levels in the overall cohort of critically ill patients. On the other hand, subgroup analysis revealed that kisspeptin levels did not correlate with the presence of sepsis and etiology of critical illness. While it is established that inflammation (e.g. in severe illness) suppresses the reproductive axis, a connection between kisspeptin and inflammation has been suggested [14]. Our study reveals a positive correlation between kisspeptin and suPAR serum levels. In accordance to the results of our study, suPAR is present in the serum, correlates to the activation level of the immune system and indicates disease severity and aggressiveness [22, 37]. Furthermore, correlation analysis showed a positive correlation between kisspeptin and interleukin-10 levels. Although there are few reports about the effects of kisspeptin on interleukin-10 activity, a study by Shirshev et al. has evaluated the effect of kisspeptin on the functional characteristics of isolated NK cells and found that kisspeptin suppresses the production of interleukin-10 [38]. However our results are in accordance with a recent study in pregnant women found that kisspeptin increases the secretion of interleukin-10 and thereby promotes the development of immunoreactivity [38]. Since it is likely that the main source of kisspeptin is not within the white blood cells we conclude that the effects of kisspeptin-stimulated interleukin-10 levels depend on the type of cells that secrete the interleukin-10. Further studies are necessary to fully investigate and understand the role of kisspeptin in critical illness and inflammation. Because a study in atria of patients transplanted for ischaemic heart disease has demonstrated lower kisspeptin levels in myocardium of patient with acute myocardial infarction [39], we tested for kisspeptin serum levels in patients with and without coronary artery disease but did not find significant differences. Nonetheless it must be noted that in the study by Maguire et al. kisspeptin was measured directly in the myocardium while the kisspeptin levels in our study were measured in serum. Hence the comparison of the results of the two studies can only be performed with restrictions.

We found a strong correlation of kisspeptin and parameters for a decreased renal function such as the glomerular filtration rate (GFR) and creatinine. In addition, we found that RRT-patients had significantly elevated kisspeptin levels at day 3. A prior study has shown that expression of kisspeptin is elevated in renal tissue of rats with chronic renal failure, pointing to a possible causal role in chronic renal impairment [40]. It is possible that the correlation of kisspeptin levels and elevated parameters of decreased renal function is caused by retention of kisspeptin in patients with an impaired glomerular filtration rate. However, based on the preexisting studies, it is also conceivable that kisspeptin has a causal role in renal disease. Functional studies in animal models are required to clarify this question.

Altogether, these results of our current study emphasize the need for further basic and translational research to understand the molecular mechanisms underlying kisspeptin up-regulation in patients with critical illness. This might serve as the basis for further exploration of kisspeptin as a biomarker and/or therapeutic target in critical illness.

## Supporting information

S1 Fig Alterations in kisspeptin expression was analyzed based on the indicated data sets. (JPG)

S2 Fig Alterations in kisspeptin serum levels in patients with coronary artery disease. Serum concentrations of kisspeptin measured at the time-point of admission to the ICU in patients with or without coronary artery disease.
(JPG)

S1 Table Correlations of IL-10 serum concentrations with that of different adipokines. (DOCX)
