# Supplementary figures and images for: Serum levels of kisspeptin are elevated in critically ill patients
Source: PLoS One. 2018 Oct 17;13(10):e0206064. doi: 10.1371/journal.pone.0206064 (PMC6192635; doi:10.1371/journal.pone.0206064)

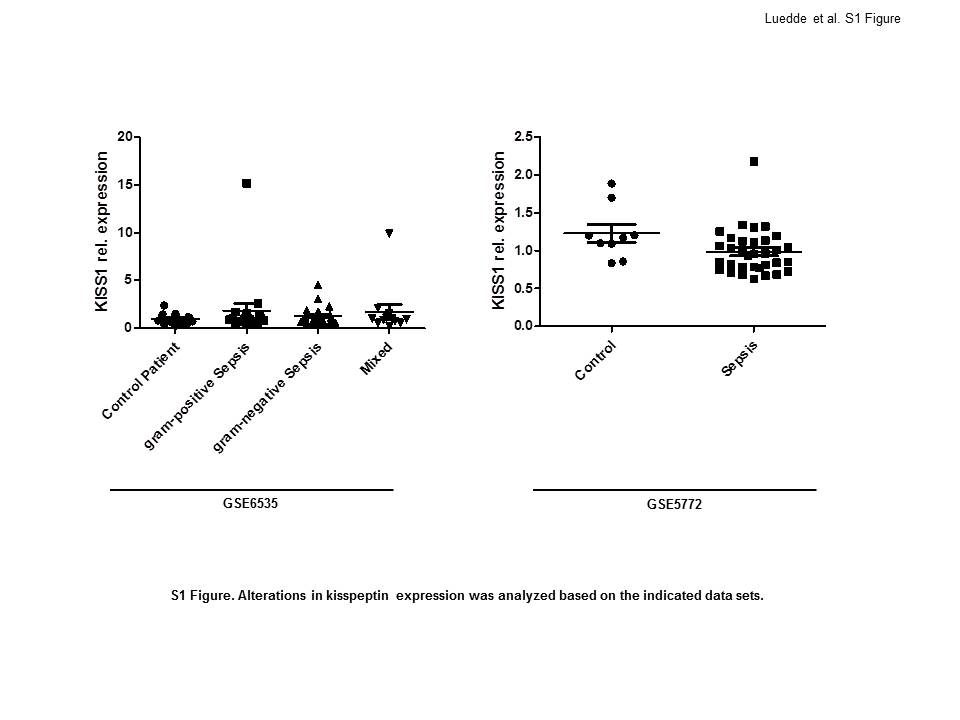

Supplement: S1 Fig — (JPG) [file pone.0206064.s001.JPG]

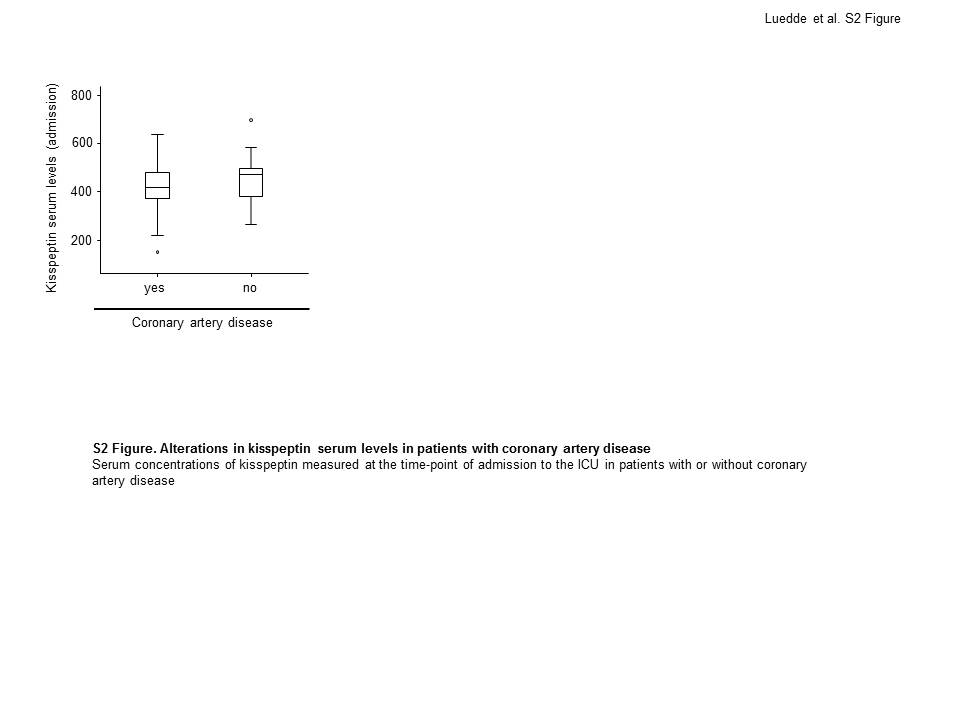

Supplement: S2 Fig — Serum concentrations of kisspeptin measured at the time-point of admission to the ICU in patients with or without coronary artery disease. (JPG) [file pone.0206064.s002.JPG]
