# Supplementary material for: Serum levels of kisspeptin are elevated in critically ill patients
Source: PLoS One. 2018 Oct 17;13(10):e0206064. doi: 10.1371/journal.pone.0206064 (PMC6192635; doi:10.1371/journal.pone.0206064)
Supplement: S1 Table — (DOCX) [file pone.0206064.s003.docx]

**S1 Table:** Correlation of IL-10 serum concentrations with that of different adipokines

|  | **ICU admission** | |
| --- | --- | --- |
| **Parameter** | **r** | **p** |
|  |  |  |
| **Kisspeptin** | **0.281** | **0.041** |
| Adiponectin | -0.053 | 0.705 |
| Leptin | -0.141 | 0.314 |
| Leptin receptor | 0,120 | 0.390 |
| Ghrelin | -0.039 | 0.784 |
| Omentin | 0.057 | 0.701 |
| **Resistin** | **0.277** | **0.044** |

r, correlation coefficient; p, p-value; r and p-values by Spearman rank correlation
